# Relationship between postoperative hypothalamic injury and water and sodium disturbance in patients with craniopharyngioma: A retrospective study of 178 cases

**DOI:** 10.3389/fendo.2022.958295

**Published:** 2022-09-02

**Authors:** Can Du, Yueshuang Leng, Quanwei Zhou, Ju-Xiong Xiao, Xian-Rui Yuan, Jian Yuan

**Affiliations:** ^1^ Department of Neurosurgery, Xiangya Hospital, Central South University, Changsha, China; ^2^ Radiological Intervention Center, Department of Radiology, Xiangya Hospital, Central South University, Changsha, China; ^3^ Department of Neurosurgery, The Institute of Skull Base Surgery and Neuro-oncology at Hunan, Changsha, China; ^4^ National Clinical Research Center for Geriatric Disorders, Xiangya Hospital, Central South University, Changsha, China

**Keywords:** diabetes insipidus, magnetic resonance imaging, hypothalamo–hypophyseal injury, craniopharyngioma, dysnatremia, postoperative complications

## Abstract

**Objective:**

To investigate the relationship between postoperative hypothalamo–hypophyseal injury (HHI) and postoperative water and sodium disturbances in patients with craniopharyngioma.

**Methods:**

The medical records, radiological data, and laboratory results of 178 patients (44 children and 134 adults) who underwent microsurgery for craniopharyngioma in a single center were reviewed. Postoperative HHI was assessed using magnetic resonance imaging. Structural defects of the hypothalamo–hypophyseal system (pituitary, pituitary stalk, floor and lateral wall of the third ventricle) were assessed in four standard T1-weighted images. The defect of each structure was assigned 1 score (0.5 for the unilateral injury of the third ventricle wall), and a HHI score was calculated.

**Results:**

The number of patients with HHI scores of 0-1, 2, 2.5-3, and >3 was 35, 49, 61, and 33, respectively. Diabetes insipidus (DI) worsened in 56 (31.5%) patients with preoperative DI, while 119 (66.9%) patients were diagnosed with new-onset DI. Hypernatremia and hyponatremia developed in 127 (71.3%) and 128 (71.9%) patients after surgery, respectively. Syndrome of inappropriate antidiuresis occurred in 97(54.5%) patients. During hospitalization, hypernatremia recurred in 33 (18.5%) patients and in 54 (35.7%) during follow-up, of which 18 (11.9%) were severe. DI persisted in 140 (78.7%) patients before discharge. No relationship was found between the HHI score and incidence of early DI, hyponatremia, syndrome of inappropriate diuretic hormone, or prolonged DI. Compared with patients with a score of 0–1, those with scores =2.5-3 (OR = 5.289, 95% CI:1.098-25.477, *P* = 0.038) and >3 (OR = 10.815, 95% CI:2.148-54.457, *P* = 0.004) had higher risk of developing recurrent hypernatremia. Patients with a score >3 had higher risk of developing severe hypernatremia during hospitalization (OR = 15.487, 95% CI:1.852-129.539, P = 0.011) and at follow-up (OR = 28.637, 95% CI:3.060-267.981, P = 0.003).

**Conclusions:**

The neuroimaging scoring scale is a simple tool to semi-quantify HHI after surgery. Recurrent and severe hypernatremia should be considered in patients with a high HHI score (>2.5). An HHI score >3 is a potential predictor of adipsic DI development. Preventive efforts should be implemented in the perioperative period to reduce the incidence of potentially catastrophic complications.

## Introduction

Craniopharyngiomas (CP) are histologically benign tumors that occur at any site along the axis from the pituitary to the hypothalamus. Surgical resection is the mainstay of treatment (1). The rate of gross total or near-total resection in the recent case series exceeded 90% (2–9). Postoperative neuroendocrine sequelae often occur because of the anatomic proximity to neurohypophyseal structures and the infiltrative growth pattern of CP (10). Water and sodium disturbances (WSDs) almost inevitably develop after surgery (11, 12). Disturbances in plasm sodium levels may cause complications, such as cerebral edema, mental disorders, lethargy, seizure, thromboembolic disease, death, and prolonged hospital stay (13, 14). Thus, intensive treatment and monitoring of fluid and electrolyte balance are crucial in patients with CP (11, 15).

The postoperative management strategies for patients after pituitary surgery have been well summarized (10, 16, 17), and many recover uneventfully despite having mild WSD. However, some patients are at higher risk of developing postoperative complications, including severe dehydration, dysnatremia, and thromboembolic disease, which can lead to disastrous results if not adequately treated.

CPs have a heterogenous growth pattern (18) with varying degrees of involvement of the neurohypophyseal system (19). Consequently, hypothalamo–hypophyseal injury (HHI) varies in each patient. The extent of HHI significantly influences the development of postoperative WSDs (10, 20, 21). Partial section or damage to the neurohypophyseal tracts may cause transient isolated hyponatremia (20). Meanwhile, severe HHI is related to permanent diabetes insipidus (DI) as well as adipsic DI (ADI) (22, 23). Therefore, we hypothesized that evaluating postoperative HHI in patients may be helpful for the individualization of treatment regimens. For sellar region tumors, risk factors for postoperative WSD have been identified (12, 24, 25). However, large case series regarding postoperative WSDs in patients with CP are uncommon. Moreover, assessing postoperative HHI and determining its relationship with postoperative WSD has not been fully explored.

In this retrospective study, we developed a neuroimaging scoring scale to perform a semi-quantitative assessment of postoperative HHI. Additionally, the relationship between HHI and WSDs was investigated. Finally, the potential clinical significance of assessing HHI was discussed.

## Clinical materials and methods

This study was approved by the Medical Ethics Committee of Xiangya Hospital, Central South University, Changsha, China. All collected data were anonymized.

### Population

A retrospective study was conducted on patients who underwent transcranial microsurgery for CP in Xiangya Hospital from July 2011 to December 2018. The surgical strategies and techniques have been summarized in our previous studies (6, 26). Briefly, the surgical goal of the chief surgeon (XR Yuan) was total resection of the tumor. The chief surgeon paid special attention to identifying pituitary stalk during the operation (26). When the tumor partially invaded the stalk, the chief surgeon dissected the tumor discretely and preserved as much of the longitudinal vasculature as possible. When the tumor extensively infiltrated the stalk, it was resected along with the tumor. If the tumor or its fragments were firmly attached to structures, such as an artery or hypothalamus, in which resection would lead to disastrous outcomes, the chief surgeon excised as much of the tumor as possible.

### Postoperative management

Our patients received intravenous (IV) fluids immediately after surgery. The fluid volume was calculated to match the intraoperative input and loss, urinary output, and physiological requirements. The most frequently used fluids were 0.9% saline, 5% dextrose water, or a mixture of fluids (half 0.9% saline and half sodium-free solution). When the pituitary stalk was resected and DI developed, desmopressin (DP) or vasopressin tannate was administered immediately via continuous aqueous infusion or intramuscular injection, respectively, as described in our previous study (27). Regular DP supplementation was administered orally when persistent DI developed. Glucocorticoids (hydrocortisone 100–200 mg IV per day during the acute postoperative period) were routinely given after surgery. According to the patient’s condition, the dosage of glucocorticoids was gradually tapered to 10–12 mg/m^2^/day before discharge. Levothyroxine replacement was initiated 2 days after surgery once adrenal insufficiency has been ruled out. Anti-epileptic therapy was not administered as prophylaxis after surgery. Daily fluid intake, urine volume, and serum electrolyte levels were monitored postoperatively in all patients. Serum electrolyte levels were measured every 12 hours for the first week; periodic measurements were performed once the urine output and serum sodium levels normalized for at least 3 days. If DI persisted on the day of discharge, patients were instructed to continue taking DP medications while adjusting doses according to the daily urine output.

### Data collection

Clinical data were collected from in-patient medical records, including admission records, progress notes, discharge summaries, nursing notes, and operation records. Biochemical results were obtained from the institution’s electronic results reporting system. All patients had a stable clinical status and underwent routine magnetic resonance imaging (MRI) examination (three-dimensional contrast-enhanced, T1-weighted, and T2-weighted MR imaging; 1.5T system, 2-mm thickness) within 48 hours after surgery. Radiological data were collected from the Picture Archiving Computer System. Data on presenting symptoms, gender, age, height, weight, tumor diameter on MRI, signs of hydrocephalus, serum sodium level during the postoperative period, surgical outcomes (pituitary stalk preservation, extent of resection), type of solution infused during the first 2 days after surgery, daily fluid intake, and urine output were gathered. Data on the follow-up serum sodium level were collected 3 months after surgery. Patients were asked to discontinue DP medications while having free access to water the day before the follow-up examinations.

The inclusion criteria were as follows: (1) patients with primary CP who underwent transcranial surgery and (2) pathologically confirmed CP. The exclusion criteria were as follows: (1) lack of continuous data on electrolytes for over 24 hours and (2) unavailable or poor-quality postoperative MR images.

Based on these criteria, 15 patients were excluded; 9 lacked continuous data on serum sodium levels, 3 could not undergo MRI because of poor clinical status, and 3 had poor-quality MR images. In total, 178 patients (44 children and 134 adults) were included in this study.

### Definition and classification criteria

**Figure 1:**
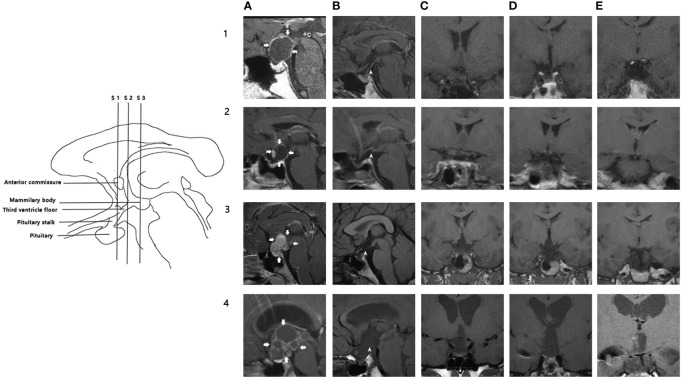
Illustration of hypothalamic injury scoring system. Left: schematic illustration of the MRI sections and anatomic landmarks used for assessment. Right: MR images in four patients (rows 1-4); five columns **(A–E)** show different sections. **(A)**: preoperative midsagittal section, arrows indicate the margin of the tumor; **(B)**: postoperative midsagittal section); **(C)**: coronal section through the anterior commissure; **(D)**: coronal section midway between the anterior commissure and mammillary bodies; **(E)**: coronal section through the mammillary bodies. 1A-1E: 1A shows a male patient with cystic supra-sellar craniopharyngioma. 1B shows pituitary, pituitary stalk (arrowhead), and third ventricle floor in this patient. Coronal sections (1C–1E) show that the third ventricle floor was intact in three sections. He had a biphasic response after surgery. HHI score: 0. Follow-up serum sodium is 142.8 mmol/L. 2A-2E: 2A shows a male patient with a mainly cystic supra-sellar tumor. (2B, 2C) show that the stalk was sectioned. Coronal sections (2C–2E) show that the third ventricle floor was intact in three sections. He had transient hypernatremia after surgery. HHI score: 1. Follow-up serum sodium: 140.3 mmol/L. 3A-3E: 3A shows a female patient with a mainly solid supra-sellar tumor involving the antero-inferior third ventricle. After surgery, defects of stalk, third ventricle floor (anterior section, medial section), and right third ventricle wall could be observed in 3B-3D. She had persistent hypernatremia after surgery. HHI score: 3.5 Serum sodium at follow-up: 148.5 mmol/L. 4A-4E: 4A shows a child with a mixed solid-cystic large supra-sellar intraventricular tumor, which caused hydrocephalus. 4B stalk and third ventricle floor were injured; pituitary was intact. 4C-4E: Defect of PS, third ventricle floor, and right third ventricle wall can be observed. HHI score: 4.5. Preoperative hydrocephalus strongly correlates with a high HHI score. WSD type: triphasic response. Follow-up serum sodium: 159.3 mmol/L. WSD, water and sodium disturbance; HIS, hypothalamic injury scoring.

The tumor was classified into four groups according to size as measured on MRI: small (<2 cm), moderate (2–4 cm), large (4–6 cm), and giant (>6 cm). Tumors seen on preoperative MRI were evaluated and categorized based on the classification proposed by Yasargil (28). We adopted an established semi-quantitative scoring system (29) to evaluate postoperative HHI. However, since blood or acute inflammation being interpreted as heterogeneous signals could lead to misinterpretation, several modifications to the original scoring system were made: (1) if the stalk was preserved but not visible on MRI, the condition of the stalk was based on the intraoperative findings; and (2) the focus was on the floor and lateral wall of the third ventricle. The pituitary gland, stalk, and floor and lateral wall of the third ventricle were assessed in four standard T1-weighted images. The selected sections are shown in **Table 1**, while the representative patients are illustrated in **Figure 1**. A defect in pituitary, stalk, bilateral third ventricle wall, each section of the third ventricle floor was assigned 1 point (0.5 point for a unilateral injury of the third ventricle wall), and the total score was calculated.

**Table 1:** MRI section used for assessment of hypothalamic injury.

Assessment of	Selected MRI section
1.Pituitary	midsagittal section
2.Pituitary stalk	midsagittal section
3.Floor of third ventricle (overview)	midsagittal section
3.1 Floor and lateral wall of third ventricle (anterior section)	coronal section through the anterior commissure
3.2 Floor and lateral wall of third ventricle (medial section)	coronal section midway between the anterior commissure and mammillary bodies
3.3 Floor and lateral wall of third ventricle (posterior section)	coronal section through the mammillary bodies

A defect in each structure was equivalent to 1 point, and the total score was calculated. According to the total score, the patients were further divided into four groups (0–1, 2, 2.5–3, >3). An independent neurosurgeon and a neuroradiologist blinded to the electrolyte levels performed the assessment. In cases of discrepancy, the two reviewers reached a consensus through discussion.

### Definition of WSDs

The following WSDs are defined as follows:

1. Hyponatremia: serum sodium level <135 mmol/L; severe hyponatremia: serum sodium level ≤125 mmol/L (30).
2. Hypernatremia: serum sodium level >145 mmol/L; severe hypernatremia: serum sodium level ≥160 mmol/L (31).
3. Preoperative DI: symptoms of polydipsia and polyuria or serum sodium>145 mmol/L. Postoperative DI: urine output >4 mL/kg/h or serum sodium >145 mmol/L and urine specific gravity <1.005 or need for vasopressin or DP administration to control urine output. Prolonged DI: DI persisting at discharge (12).
4. Syndrome of inappropriate antidiuresis (SIAD) secretion: oliguria (<2 mL/kg/h) associated with hyponatremia (<135 mmol/L) in the absence of prerenal or renal failure, diluting effects of fluid replacement, or gluco- or mineralocorticoid deficiency (12).

### Statistical analysis

All analyses were performed using the Statistical Package for Social Science (SPSS version 22). Descriptive statistics were calculated for continuous variables, while frequency distributions were obtained for categorical data. Categorical variables in different groups were analyzed using the Chi-square test or Fisher’s exact test as appropriate. The Kruskal–Wallis test was used to compare the continuous variables between groups. Variables without collinearity and *P* value ≤ 0.2 were selected for logistic regression analysis. Binary logistic regression analysis was performed to evaluate risk factors for binary outcomes. Hosmer and Lemeshow test was conducted to assess the goodness of fit of the regression models. *P* values <0.05 were considered statistically significant, and all tests were two-sided.

## Results

### Patient demographics and study characteristics

In total, 178 patients (44 children and 134 adults) were included in this study. The clinical presentation and radiological characteristics of patients on admission are summarized in **Table 2**. Preoperative DI presented in 56 patients, and 13 of them had hypernatremia. All patients had normal renal function. The mean tumor size was larger in children (3.80 ± 0.81 vs. 3.25 ± 1.06 cm, *P* = 0.002). The incidence of hydrocephalus was also higher in children (*P* = 0.004). Total or near-total resection (only small tumor fragments remained) was achieved in 165 (92.7%) cases, and no patients received adjuvant therapy. The longitudinal vasculature of the stalk was totally and partially preserved in 15 and 7 patients, respectively. No death was observed within the first 30 days after surgery. Deterioration of consciousness occurred in 19 patients (4 children and 15 adults) after surgery; 18 regained consciousness before discharge, and one died two months after surgery.

**Table 2:** Clinical and radiological characteristics.

Characteristics		Children (N=44)	Adults (N=134)
Male sex-no. (%)		30(68)	80(60)
Presenting symptoms-no. (%)			
	Headaches	28 (64)	124 (93)
	Vision deficits	18 (41)	99 (74)
	Polydipsia and polyuria	14 (32)	43 (32)
	Hypothalamic symptoms	5 (11)	16 (12)
	Growth retardation	14 (32)	
	Menstrual disorder		19 (14)
	Decreased libido		24 (18)
Mean tumor diameter(cm)		3.80 ± 0.81	3.25 ± 1.06
Tumor size-no. (%)			
	Small	1 (2)	13 (10)
	Median	23 (52)	85 (63)
	Large	18 (41)	34 (25)
	Giant	0	4 (3)
Yasargil classification			
	Intrasellar infradiaphragmatic	0	2 (1.5)
	Intra-/supra-sellar and intra/supradiagphragmatic	14 (31.8)	32 (24.6)
	Supradiagphragmatic extraventricular (compressing third ventricle floor)	7 (15.9)	49 (37.7)
	Intra- and extraventricular	23 (52.3)	24 (18.5)
	paraventricular	0	22 (16.9)
	Purely intraventricular	0	1 (0.8)
Hydrocephalus-no. (%)		21 (47.7)	28 (20.9)
Pituitary stalk preservation-no. (%)		5 (11)	17 (13)
Total resection-no. (%)		42 (95)	123 (92)

### Postoperative HHI

Our results showed that defects in the hypothalamo–hypophyseal structure were very common; 95% of patients had HH defects observed on MRI. Meanwhile, only one child and six adults had an HHI score of 0. The details of the patients’ HHI scores were listed in **Table 3**. The score was not different among children and adults. The stalk was the most frequently affected structure (87.6%, 155/178), followed by the third ventricle floor between the anterior commissure and mammillary bodies (61.8%,110/178). The proportion of patients with pituitary gland and third ventricle floor injury (mammillary body section) was higher in children.

**Table 3:** Assessment of hypothalamo–hypophyseal injuries on MRI stratified according to age group and HI score.

		HI score of children (N=44)				HI score of adults (N=134)				
		0-1(n=6)	2(n=14)	2.5-3(n=13)	>3(n=11)	0-1(n=29)	2(n=35)	2.5-3(n=48)	>3(n=22)	NS*
Assess of injuries on MRI-no. (%)										
	Pituitary	0 (0)	9 (64.3)	1 (7.7)	2 (18.2)	3 (10.3)	4 (11.4)	3 (6.3)	4 (18.2)	
	Pituitary stalk	3 (50)	12 (85.7)	13 (100)	11 (100)	17 (58.6)	30 (85.7)	48 (100)	22 (100)	
Third ventricle floor-no. (%)										
	Anterior commissure section	0	2 (14.3)	12 (92.3)	11 (100)	0	14 (40)	39 (81.3)	21 (95.5)	
	Between anterior commissure and mammillary body	2 (33.3)	2 (14.3)	10 (76.9)	11 (100)	2 (6.9)	19 (54.3)	42 (87.5)	22 (100)	
	Mammillary body section	0	0	1 (7.7)	9 (81.8)	0	0	2 (4.2)	11 (50)	
	Third ventricle wall (unilateral/bilateral)	0/0	0/0	1 (7.7)/0	3 (27.3)/0	1 (3.4)/0	2 (5.7)/0	8 (16.7)/3 (6.3)	9 (40.9)/7 (31.8)	

*Chi-square test or Fisher’s exact test, as appropriate.

The HHI score was significantly correlated with tumor location, size, and presence of hydrocephalus (**Table 4**). Patients with infra- or supradiaphragmatic tumors usually had lower scores; only five (10.4%) patients with these tumors had scores between 2.5 and 3. The proportion of patients with a score ≥2.5 was higher in those who had tumors with third ventricle involvement, which increased to 48.2% in patients with a supra-sellar tumor compressing the third ventricle floor. In contrast, the percentage of patients with a score ≥2.5 was 91.8% in those with extra- and intraventricular tumors and 77.3% in those with paraventricular tumors. In patients with hydrocephalus, 91.8% had a score ≥2.5 (illustrated in **Figure 1**, case 4).

**Table 4:** Relation between preoperative tumor features and hypothalamic injury after surgery.

		Hypothalamic injury score				
		0-1	2	2.5-3	3.5-5		
Total number		35	49	61	33	
Yasargil classification						χ^2 =^ 87.337; df=18; *P*<0.001*
	Intrasellar infradiaphragmatic	0	2	0	0	
	Intra-/supra-sellar and intra/supradiagphragmatic	22	21	5	0	
	Supradiagphragmatic extraventricular (compressing third ventricle floor)	12	17	18	9	
	Intra- and extraventricular	1	3	26	19	
	paraventricular	0	5	12	5	
	Purely intraventricular	0	1	0	0	
hydrocephalus		1	3	25	20	χ^2 =^ 46.69; df=3; *P*<0.001*
Tumor size-no. (%)						χ^2 =^ 40.675; df=9; *P*<0.001*
	Small (<2 cm)	9	5	0	0	
	Median (2-4 cm)	24	32	38	14	
	Large (4-6 cm)	2	11	21	18	
	Giant (>6 cm)	0	1	2	1	

### Rate and course of WSDs

DI worsened in 56 patients with preoperative polyuria and polydipsia, while 119 patients were diagnosed with new-onset DI. The incidence of postoperative DI was 98.3%. DI usually developed within the first several hours after surgery. Antidiuretic hormone treatment was initiated in 88% of the patients on the day of surgery. Hypernatremia was the most common electrolyte disorder in the early postoperative stage, occurring in 127 (71.3%) patients. Hypernatremia was noted in 70 (55.1%) individuals on the operation day and in 23 (18.1%) on the first postoperative day. The median duration of hypernatremia was 1 day (interquartile range, 1–3 days). The HHI score correlated with the occurrence of hypernatremia (*P* = 0.021) but not with DI. Hypernatremia tended to occur less in adults (OR = 0.404, 95% CI: 0.161–1.012, *P* = 0.043). Patients with a score >3 were at risk to develop hypernatremia, albeit without significance (OR = 3.604, 95% CI: 0.884–14.698, *P* = 0.074). Severe hypernatremia was noted in 19 patients, and the median onset was 1 day after surgery (interquartile range, 1–3 days). Patients with a score >3 were at significantly increased risk for developing severe hypernatremia (OR = 15.487, 95% CI: 1.852–129.539, *P* = 0.011).

Hyponatremia occurred in 128 (71.9%) patients; of these, 125 developed early DI before the onset of hyponatremia. Hyponatremia usually occurred later than hypernatremia; the median onset was 4.5 days after surgery (interquartile range, 3–6 days), and the median duration was 3 days (interquartile range, 1.25–4). Severe hyponatremia occurred in 9 children and 20 adults. The median onset of severe hyponatremia was 6 days after surgery (interquartile range, 5–7 days). Four children had epileptic seizures, and 17 patients had symptoms such as headache, nausea, loss of appetite, and weakness. In 97 patients, the cause of hyponatremia was considered to be SIAD. No association was found between the HHI scores and the incidence of hyponatremia or SIAD.

**Figure 2:**
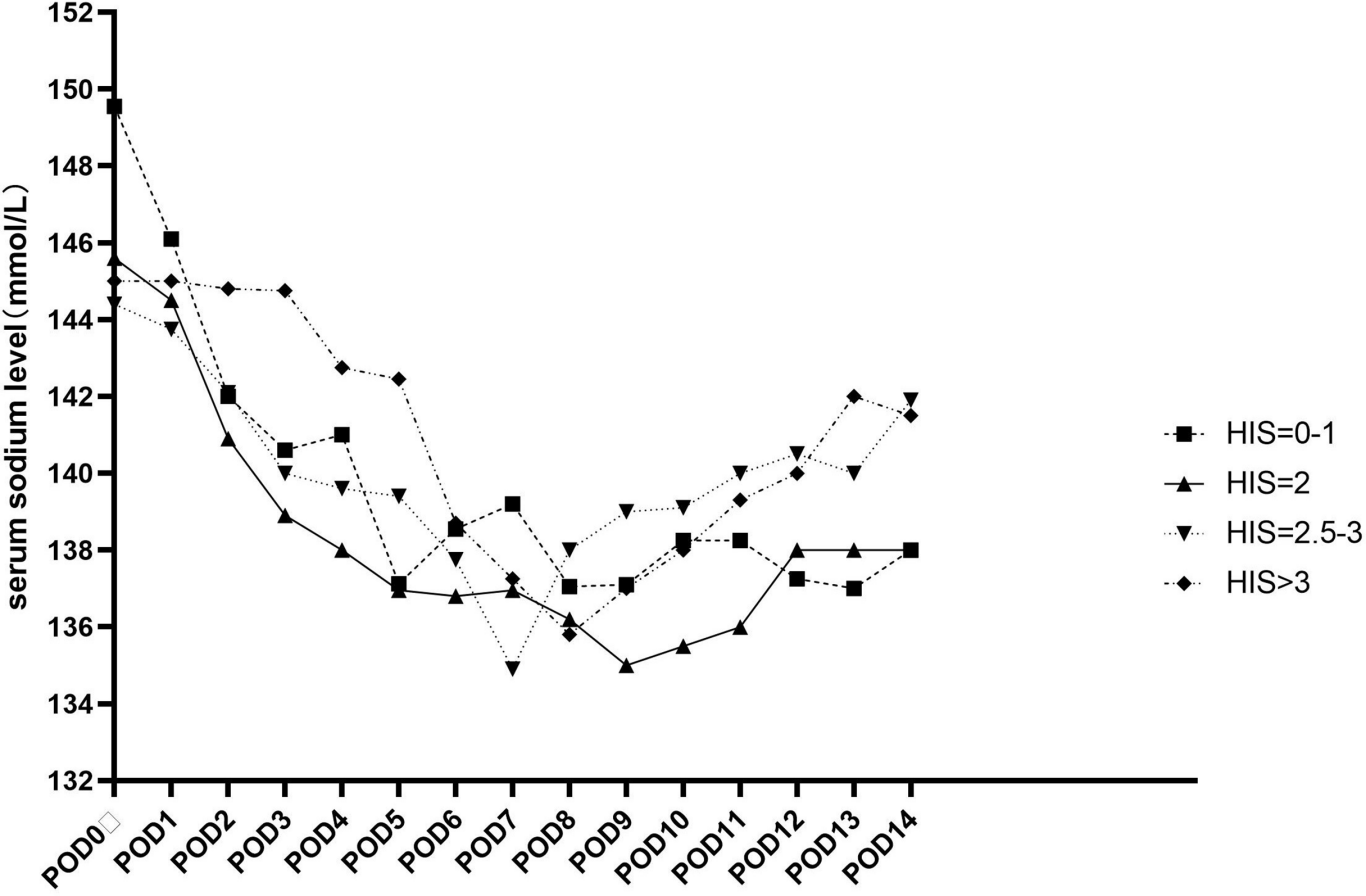
The Median serum sodium value of patients had different HI score.

Upon discharge, 140 patients still had DI and needed DP supplementation. The HHI score was not correlated with prolonged DI. Hypernatremia recurred in 33 patients during hospitalization. The median onset of recurrence was 10 days after surgery (interquartile range, 7.5–13.5). Despite receiving antidiuretic therapy, hypernatremia tended to recur more frequently in patients with a score of 2.5–3 and >3 than in those with a score of 0–1 (*P* = 0.001). The median serum sodium level in patients with different scores are shown in **Figure 2**.

**Figure 3:**
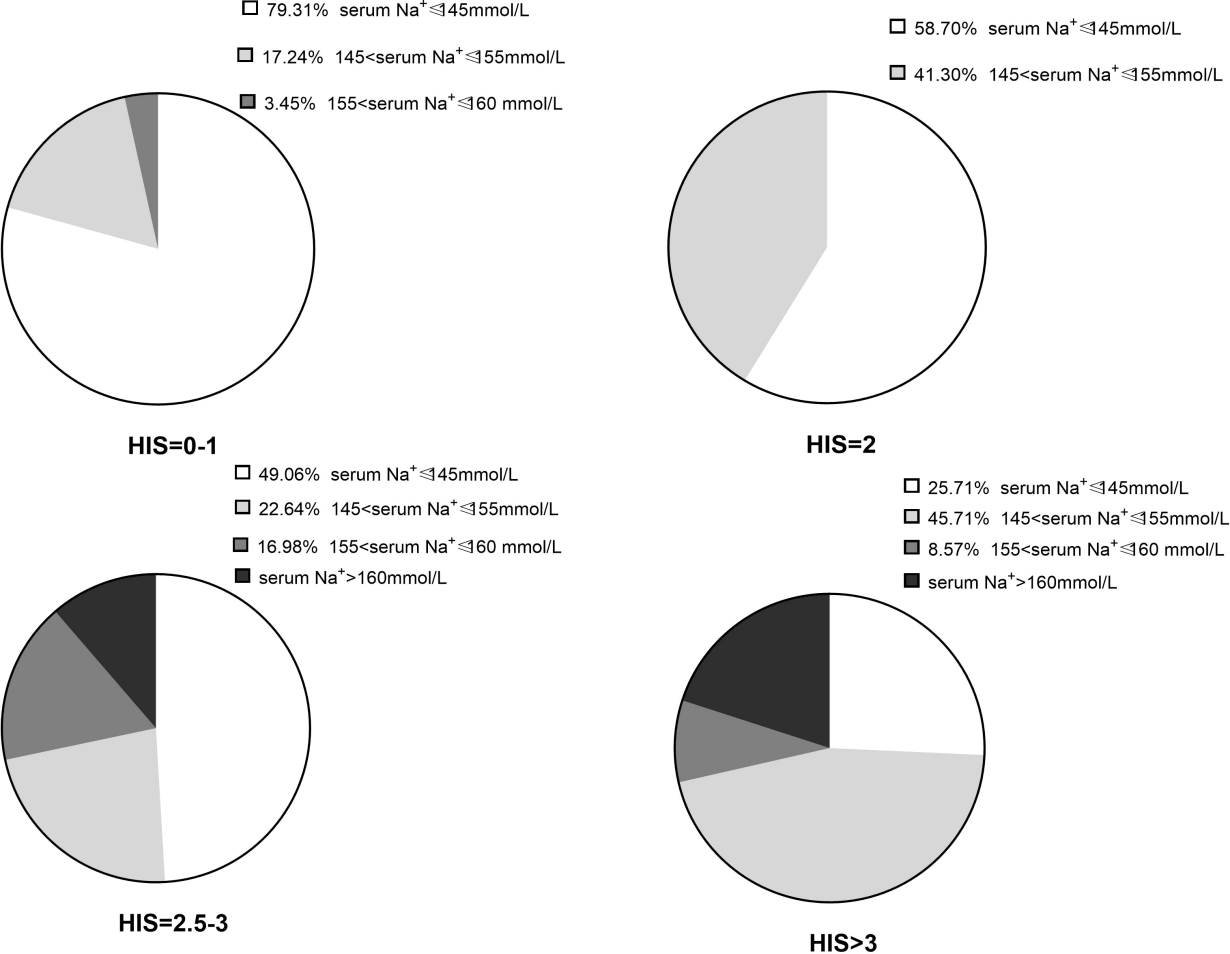
Serum sodium results of patients had different HI scores on follow-up day.

Follow-up examination data were retrieved from 151 patients. The serum sodium levels were normal and elevated in 97 and 54 patients, respectively. Of the 54 patients, 18 (11.9%) had severe hypernatremia. The serum sodium levels are shown in **Figure 3**. Among the patients who had normal serum sodium levels, we noted two different subgroups. After the discontinuation of DP supplementation, serum sodium levels and urine specific gravity remained normal in the first subgroup; of these patients, 44.8% (13/29), 34% (14/41), 25.5% (13/51), and 7.4% (2/27) had an HHI score of 0–1, 2, 2.5–3, and >3, respectively. The second subgroup showed compensation ability to maintain normal serum sodium level, despite low urine specific gravity and urine-to-serum osmolality still presented. Of these patients, 48.3% (14/29), 41.5% (17/41), 31.4% (16/51), and 29.6% (8/27) had a score of 0–1, 2, 2.5–3, and >3, respectively. The risk of hypernatremia at follow-up correlated with the HI score; patients with a score >3 had a significant risk of developing severe hypernatremia (OR = 28.637, 95% CI: 3.060–267.981, *P* = 0.003). All statistical results are listed in **Table 5** and the **Supplementary Table**.

**Table 5:** Statistics between hypothalamic injury score (HIS) and WSD.

	Hypothalamic injury score(%)				Statistics
		0-1 (n = 35)	2 (n = 49)	2.5-3 (n = 60)	3.5-5 (n = 33)	
Early DI	34	48	60	33	NS*
Institute antidiuretic therapy	31 (88.6)	47 (95.9)	60 (98)	31 (93.9)	NS*
Postoperative hypernatremia-no. (%)	25 (71.4)	29 (59.2)	45 (72.6)	30 (93.8)	χ^2 =^ 9.734; df=3; *P*=0.021*
Median onset time of hypernatremia-(postoperative day)	0	0	0	1	NS*
Median duration of hypernatremia-(days)	1	1	1	1	NS§
Severe hypernatremia during hospital-no. (%)	1(2.9)	6 (12.2)	7 (11.3)	11 (34.4)	χ^2 =^ 11.674; df=3; *P*=0.008*
Postoperative hyponatremia-no. (%)	27 (77.1)	35 (71.4)	42 (67.7)	24 (75.0)	NS*
SIAD	24 (88.9)	22 (62.9)	34 (80.9)	17 (70.8)	NS*
Severe hyponatremia-no. (%)	7 (20.0)	10 (20.4)	8 (12.9)	5 (15.6)	NS*
Median onset time of hyponatremia-(postoperative day)	4	5	4	5	NS*
Median duration of hyponatremia-(days)	2.5	2	2	2	NS§
Prolonged DI	25 (71.4)	35 (71.4)	50 (83.3)	30 (90.9)	NS*
Triphasic response -no. (%)	20 (57.1)	21 (42.85)	34 (55.7)	16 (48.5)	NS*
Recurrent hypernatremia during hospitalization-no. (%)#	2 (5.7)	4 (8.2)	14 (23)	13 (39.4)	χ^2 =^ 17.594; df=3; *P*=0.001*
Recurrent hypernatremia at follow-up-no. (%)#	2 (6.9)	13 (28.9)	22 (41.5)	17 (60.7)	χ^2 =^ 20.930; df=3; *P*=0.000*
Serum Na^+^ at follow-up(mmol/l)	142.9± 3.5	144.54 ± 4.3	146.9 ± 7.1	151.7 ± 9.7	P<0.001§

*Chi-square test or Fisher’s exact test, as appropriate.

Follow up data was available in 151 patients.

§Kruskal–Wallis test.

NS, no significance.

## Discussion

Patients would face a series of postoperative neuroendocrine sequelae, and WSDs almost inevitablely develop after total tumor removal (11, 12). Aside from intensive treatment and daily monitoring, a better understanding of the incidence and course of WSDs is also important for patients with CP. Previous studies have suggested that the postoperative course of WSDs varies among patients with different degrees of HHI (20, 32, 33). In this study, we used a neuroimaging scoring scale to assess HHI and investigated the relationship between the HHI score and various types of WSDs, such as DI, SIAD, hypernatremia, hyponatremia, and recurrent hypernatremia. Our results show that there is no relationship between the HHI score and incidence of early DI, prolonged DI, SIAD, and hyponatremia. However, patients with an HHI score ≥2.5 had a higher risk of developing recurrent and severe hypernatremia, with median onsets of 10 days after surgery and between discharge and follow-up, respectively. The results suggest that assessing HHI may provide important information for implementing individualized treatment regimens in these patients.

The rate of DI is higher in CP than other pituitary diseases (11, 25), and DI occurs more frequently in patients who underwent gross total resection(GTR) (3, 6–9, 26). In a early review, permanent DI occurs in 80-93% patients after complete resection (1). In recent studies, the reported DI rate differed widely. Shi et al. reported a large cohort comprising 1053 patients with a 89.6% GTR rate. The overall DI rate was 32.8% (31.5% for stalk preservation group, and 68.5% for unidentified group) (4). Qi et al. compared postoperative DI rate of different tumor type. The overall DI rate was 70.9%, and the DI rate was 69.7%, 48.3%, and 80.6% in type Q, type S, and type T, respectively (34). Qi et al. reported permanent DI rate was 52.6% in transcranial surgery group and 50.4% in endoscopic endonasal surgery group (8). Nie et al. found endoscopic endonasal surgery group had higher GTR rate(89.8%) and less DI rate(51.1%) compared with transcranial surgery group(GTR rate 77.8%, DI rate 72.4%) (7). Yamada et al. reported a 83% DI rate in primary surgery group (3). However, most of these studies didn’t document details such as strategy of managing stalk, specific definition of DI, and rate of antidiuretic hormone supplement, making it less robust to compare the results directly. Ogawa et al. reported all patients needed antidiuretic therapy after stalk sectioning (2). In a recent study analyzing surgical results of preserving stalk, the author reported a 95% DI rate in stalk sacrificed group and a 50% DI rate in stalk preservation group after surgery (9). Overall, the postoperative DI rate is largely determined by surgical strategy and the specific definition in each study.

The rate of early DI in this study was comparable to previous studies (2, 9). The high DI rates in this study can be attributed to two factors. The first is our surgical goal. Similar to other studies (2, 4, 34), the chief surgeon believe the goal of surgery should be complete tumor removal with stalk preservation. However, if the stalk appears damaged by the tumor, the patient may be better served by having the stalk sacrificed to ensure GTR (9). Stalk preservation led to a GTR rate of only 73%, with an 18% progression rate and a 9% recurrence rate (9). Our previous study identified tumor invasion in all the resected stalk (26). Compared with intentional subtotal resection, damage to the neurohypophyseal structures can be caused inevitably by intraoperative dissection, leading to DI, irrespective of the final extent of excision (12). The other factor was our postoperative management strategy and the definition of DI in this study. Patients who received DP medication were classified as DI in our study. Antidiuretic treatment was instituted immediately since we were vigilant about the sudden increase in urine output. Moreover, given the high rates of DI after stalk resection, we instituted antidiuretic treatment preventatively in patients whose stalk was sacrificed. Therefore, patients who had transient DI were also included. In our study, the rate of patients who received DP medication decreased to 78.7% before discharge. Therefore, we believe the results of this study are generalizable to patients underwent intended total resection of CP.

Hyponatremia is a common dysnatremia in patients who undergo surgery for pituitary lesions (34). The median onset of hyponatremia is usually later than that of DI (16, 34). In our study, hyponatremia occurred after DI in approximately 70% of patients. Age, gender, tumor size, rate of decline of serum sodium level, Cushing’s disease, and preoperative hyponatremia were reported as possible predictors of hyponatremia in patients with pituitary adenoma (35, 36). However, in CP, predictors of hyponatremia remain inconclusive. Hyponatremia may be caused by certain conditions, such as SIAD, glucocorticoid insufficiency, excessive fluid infusion, and cerebral salt wasting syndrome, which may occur in isolation or combination (11). Determining the exact cause of hyponatremia in each patient is difficult in a retrospective study (15, 24). In this study, 75.8% of the patients met the SIAD criteria. SIAD can be induced by endogenous release of arginine vasopressin (AVP) or exogenous causes, such as DP supplements. These two factors can coexist in patients who underwent surgeries for pituitary tumors and needed DP to control DI. Besides, large inter-individual differences in response to DP medication may exist (33), which makes it difficult to judge if SIAD was induced by endogenous AVP or DP medication. These factors, which are difficult to be quantitatively assessed in a retrospective study, may have a strong influence on the occurrence of hyponatremia. Given the high incidence of the various types of WSD and the shift from one type to another, close monitoring and adequate adjustments are required during the first postoperative week when treating patients who underwent surgery for CP.

Compared with other pituitary lesions, CP is associated with prolonged or permanent DI (33). This occurs when all stored AVP is depleted, and 80–90% of AVP synthesizing neurons undergo degeneration (37). The incidence of prolonged or permanent DI is between 14.2% and 50% in patients with CP (8, 9, 12, 38). In this study, the incidence of prolonged DI was not different among the patient groups. However, similar to early-phase DI, hypernatremia developed more frequently in patients with an HHI score >2.5 despite receiving antidiuretic treatment. The specific mechanism behind this phenomenon remains unclear. In patients who underwent transcranial hypothalamic surgery, serum AVP immunoreactivity was high, but the AVP was not bioactive and greatly attenuated the antidiuretic response to standard AVP treatment (39). Another possible mechanism was the severity of vasopressin deficiency, which could lead to the downregulation of the synthesis of aquaporin-2 water channels in the principal cells in the collecting ducts of the kidney, causing secondary nephrogenic DI (40).

Spontaneous resolution from DI in patients who underwent pituitary stalk transection has been reported (2, 41, 42); its rates range from 44.4% to 53.7%, similar to that in patients with an HHI score of 0–1 in this study. Among the patients who experienced spontaneous DI resolution, 60% discontinued antidiuretic hormone supplementation within 10 weeks, and 80% discontinued medication within 30 weeks (2). Axonal damage near neurons could cause more severe degeneration. Stalk disturbances at its proximal portion near the hypothalamus were associated with more severe postoperative DI compared with disturbances at the distal portion near the posterior lobe of the pituitary gland (41, 43). This may explain why patients with high scores had lesser rates of spontaneous DI resolution. We also found that maintaining sodium homeostasis was impaired in patients with high scores. When osmoreceptors work normally, the loss of body water causes a rise in serum osmolality that stimulates thirst and induces compensatory polydipsia. The resulting increase in water intake restores balance with urine output and stabilizes the osmolality of body fluids at a new and slightly higher, but still normal, level (33). Adipsic DI is one of the most severe forms of central DI and is characterized by an inappropriate lack of thirst and consequent failure to drink to correct hyperosmolality (22, 44). Adipsic DI should be suspected in conscious patients presenting with severe hypernatremia (45). Patients with adipsic DI are at high risk for potentially lethal complications, including hypernatremia, dehydration, venous thromboembolism, and seizures. Adipsic DI prevention requires a series of measures, including regular DP supplementation to control urine output, consumption of approximately 2 L of fluids daily, and body weight monitoring to detect changes in fluid balance (22). Patient education regarding the treatment of adipsic DI and symptoms consistent with the onset of dysnatremia should be performed during the hospitalization of these patients. Our results suggest that the neuroimaging scoring scale could be a screening tool to determine patients at risk of developing ADI. Clinicians may confirm the presence of adipsic DI in these patients and educate them regarding the management of adipsic DI. By identifying patients at risk for developing adipsic DI, preventive efforts can be implemented in the perioperative setting to reduce the incidence of potentially catastrophic complications.

The scoring scale in this study is simple and reproducible in most medical centers. It assesses HHI in patients using anatomical landmarks on sagittal and coronal MRI slices. This approach did not employ volumetric assessments and measurements, which are difficult to compare between patients (29). We also evaluated the relationship between the types of CP based on preoperative MRI (using the Yasargil classification (28)) and WSDs. Although there is an association between the types of craniopharyngioma associated with WSD, such as recurrent hypernatremia, the correlation was not as strong as that with the HHI score. Additionally, differentiation between extra-axial CP distorting the hypothalamus and tumors originating from the infundibulo-tuberal area via preoperative MRI is difficult (46). The anatomical relationship of the CP to the hypothalamus may appear similar on preoperative MRI, but the postoperative HHI may be very different. At last, this scoring system can be adopted by clinicians unfamiliar with the preoperative radiological characteristics of CP but who deal with patients with CP.

Recently, endoscopic transsphenoidal surgery for CP has gained popularity worldwide (3, 7–9). However, the number of patients who underwent transsphenoidal surgery was not comparable to patients who underwent transcranial surgery in our center; thus, they were excluded from the study. We believe that assessing for postoperative HHI also holds clinical value for patients who underwent transsphenoidal surgery because the HHI incidence following CP resection via transsphenoidal surgery is high in these cohorts. Patients had comparable results in transcranial surgery group and endoscopic endonasal surgery group, in terms of rate of permanent DI, and hypothalamic status (8). Furthermore, an endoscopic transsphenoidal approach offers a better surgical view under the optic chiasm, allowing for closer observation of the hypophyseal structures. This could be a complementary method to assess HHI more precisely.

The strength of this study is the large sample size and complete radiological images and electrolyte data. A single experienced neurosurgeon performed all the surgeries, and all patients received relatively uniform management after surgery. However, this study has some limitations. Firstly, our data were collected retrospectively, and some records were incomplete. Additionally, we did not perform examinations to determine the incidence of adipsic DI in patients with high HHI scores. Secondly, only patients who underwent transcranial microsurgery were included. In the future, examinations such as the measurement of thirst ratings (47) and plasma AVP concentration during intravenous infusion of hypertonic saline (48) should be carried out to identify the incidence of adipsic DI in patients with a high HHI score. The efficacy of preventive treatment in these patients should also be evaluated. Further studies on patients who underwent transsphenoidal surgery for CP are also necessary.

## Conclusion

The neuroimaging scoring scale is a simple tool to semi-quantify HHI after surgery. The development of recurrent and severe hypernatremia should be considered in patients with a high HHI score (≥2.5). An HHI score >3 is a potential predictor of adipsic DI development. Preventive efforts can be implemented in the perioperative setting to reduce the incidence of potentially catastrophic complications.

## Data availability statement

The original contributions presented in the study are included in the article/**Supplementary Material**. Further inquiries can be directed to the corresponding author.

## Ethics statement

The studies involving human participants were reviewed and approved by Medical Ethics Committee of Xiangya Hospital, Central South University, Changsha, China. Written informed consent to participate in this study was provided by the participants’ legal guardian/next of kin.

## Author contributions

CD and JY conceived the project. X-RY performed most of the surgery for patients in this study. CD and YL performed radiological assessment of all the subjects. QZ and J-XX collected data. CD, YL and QZ analyzed the data. CD and JY wrote the manuscript. All authors contributed to the article and approved the submitted version.

## Funding

This work was supported by the Hunan Provincial Natural Science Foundation of China (No. 2021JJ41050).

## Acknowledgments

We would like to thank Carlos Benjamin (Editage) for English language editing and Dr. Chengyuan Feng for his suggestion to this study.

## Conflict of interest

The authors declare that the research was conducted in the absence of any commercial or financial relationships that could be construed as a potential conflict of interest

## Publisher’s note

All claims expressed in this article are solely those of the authors and do not necessarily represent those of their affiliated organizations, or those of the publisher, the editors and the reviewers. Any product that may be evaluated in this article, or claim that may be made by its manufacturer, is not guaranteed or endorsed by the publisher.
