# Supplementary material for: Relationship between postoperative hypothalamic injury and water and sodium disturbance in patients with craniopharyngioma: A retrospective study of 178 cases
Source: Front Endocrinol (Lausanne). 2022 Sep 2;13:958295. doi: 10.3389/fendo.2022.958295 (PMC9478176; doi:10.3389/fendo.2022.958295)
Supplement: Supplementary file 1 [file Table_1.docx]

| **Supplemental Table 1. Univariate and Multivariate Analyses of Factors Associated with Severe Serum Sodium Abnormalities** | | | | | | |
| --- | --- | --- | --- | --- | --- | --- |
|  | Univariate Analyses | | | Multivariate Analyses | | |
|  | Variable | OR(95%) | P | OR(95%) | P | Hosmer and Lemeshow tests |
| Hypernatremia | **Age (children vs adult)** | **0.414(0.171-1.066)** | **0.051** | 0.404(0.161-1.012) | 0.053 | χ^2^=9.357; df=8; *P*=0.313 |
|  | Sex (Female vs male) | 1.266(0.648-2.471) | 0.490 |  |  |  |
|  | Body mass index(<25 vs ≥25) | 1.591(0.699-3.620) | 0.269 |  |  |  |
|  | **HI score baseline (score=0-1)** |  | **0.031** |  | 0.028 |  |
|  | Score=2 | 0.580(0.229-1.468) | 0.250 | 0.520(0.202-1.339) | 0.175 |  |
|  | Score=2.5-3 | 1.125(0.444-2.849) | 0.804 | 1.091(0.427-2.787) | 0.855 |  |
|  | **Score>3** | **4(0.991-16.142)** | **0.051** | **3.604(0.884-14.698)** | **0.074** |  |
|  | Total resection VS subtotal or partial resection | 2.191(0.468-10.261) | 0.320 |  |  |  |
| Severe hypernatremia | Age (children vs adult) | 0.429  (0.177-1.040) | 0.061 | 0.495(0.195-1.253) | 0.138 | χ^2^=10.005; df=7; *P*=0.188 |
|  | Sex (Female vs male) | 0.941(0.397-2.233) | 0.891 |  |  |  |
|  | Body mass index(<25vs≥25) | 1.264(0.489-3.267) | 0.629 |  |  |  |
|  | HI score baseline (score=0-1) |  |  |  | **0.014** |  |
|  | Score=2 | 4.744(0.545-41.313) | 0.159 | 4.369(0.498-38.330) | 0.183 |  |
|  | Score=2.5-3 | 4.407(0.519-37.415) | 0.174 | 4.296(0.504-36.632) | 0.183 |  |
|  | **Score>3** | **17(2.048-141.091)** | **0.009** | **15.487(1.852-129.539)** | **0.011** |  |
|  | Total resection VS subtotal or partial resection | 0 | 0.999 |  |  |  |
| Recurrent hypernatremia during hospitalization |  |  |  |  |  | χ^2^=2.811; df=7; *P*=0.902 |
|  | Age (children vs adult) | 0.496 (0.220-1.115) | 0.09 | 0.591 (0.231-1.509) | 0.271 |  |
|  | Sex (Female vs male) | 1.578(0.700-3.558) | 0.272 |  |  |  |
|  | Body mass index(<25 vs ≥ 25) | 0.815(0.326-2.037) | 0.662 |  |  |  |
|  | **HI score baseline (score=0-1)** |  | **0.002** |  | **0.002** |  |
|  | Score=2 | 1.467(0.253-8.489) | 0.669 | 1.428(0.240-8.501) | 0.696 |  |
|  | **Score=2.5-3** | **4.915(1.046-23.087)** | **0.044** | **5.289(1.098-25.477)** | **0.038** |  |
|  | **Score>3** | **10.725(2.189-52.537)** | **0.003** | **10.815(2.148-54.457)** | **0.004** |  |
|  | Total resection VS subtotal or partial resection | 0 | 0.999 |  |  |  |
| Hypernatremia at follow-up |  |  |  |  |  | χ^2^=6.420; df=8; *P*=0.600 |
|  | **Age (children vs adult)** | **0.529(0.263-1.063)** | **0.074** | 0.578(0.273-1.224) | 0.152 |  |
|  | Sex (Female vs male) | 0.868(0.461-1.634) | 0.661 |  |  |  |
|  | Body mass index(<25vs≥25) | 1.185(0.580-2.421) | 0.641 |  |  |  |
|  | **HI score baseline (score=0-1)** |  | **0.000** |  | 0.001 |  |
|  | **Score=2** | **4.269(1.122-16.228)** | **0.033** | **4.034(1.054-15.432)** | **0.042** |  |
|  | **Score=2.5-3** | **6.919(1.904-25.138)** | **0.003** | **6.863(1.882-25.030)** | **0.004** |  |
|  | **Score>3** | **16.410(4.153-64.844)** | **0.000** | **15.427(3.882-61.307)** | **0.000** |  |
|  | Total resection vs subtotal or partial resection | 1.187(0.350-4.025) | 0.783 |  |  |  |
| Severe hypernatremia at follow-up |  |  |  |  |  | χ^2^=3.002; df=8; *P*=0.934 |
|  | Age(children vs adult) | 0.484(0.173-1.350) | 0.166 |  |  |  |
|  | Sex (Female vs male) | 0.347(0.126-0.954) | 0.040 | 0.277(0.068-0.751) | 0.015 |  |
|  | Body mass index(<25 s≥25) | 0.624(0.170-2.291) | 0.477 |  |  |  |
|  | **HI score baseline (score=0-1)** |  | 0.006 |  | 0.007 |  |
|  | Score=2 | 0 | 0.998 | 0 | 0.998 |  |
|  | Score=2.5-3 | 3.652(0.418-31.939) | 0.242 | 5.416(0.593-49.473) | 0.134 |  |
|  | **Score>3** | **19.250(2.271-163.170)** | **0.007** | **28.637(3.060-267.981)** | **0.003** |  |
|  | Total resection VS subtotal or partial resection | 2.822(0.688-11.582) | 0.150 |  |  |  |
